# A retrospective study on the characteristics of renal pathological grades in HSPN children with mild to moderate proteinuria

**DOI:** 10.3389/fped.2022.1029520

**Published:** 2022-11-18

**Authors:** Yan Cao, Tian Shen, Yongzhen Li, Lanjun Shuai, Qiaoping Chen, Shuanghong Mo, Canlin Li, Xiaoyan Li, Ying Wang, Xiaochuan Wu

**Affiliations:** ^1^ Department of Pediatrics, The Second Xiangya Hospital, Central South University, Changsha, China; ^2^ Department of Digestive Nutrition, Hunan Children’s Hospital, Changsha, China

**Keywords:** children, Henoch–Schönlein purpura nephritis, proteinuria, renal pathology, immune complexes

## Abstract

**Objective:**

To investigate the characteristics of renal pathological grades in Henoch–Schönlein purpura nephritis (HSPN) children with mild to moderate proteinuria and the correlation between pathological grade and severity of proteinuria among this population.

**Methods:**

HSPN children who were presented with mild (150 mg <24 h urinary protein <25 mg/kg) to moderate (25 mg/kg ≤24 h urinary protein <50 mg/kg) proteinuria and performed renal biopsy without steroid ± immunosuppressant treatment in the Second Xiangya Hospital between January 2010 and March 2021 were involved. We retrospectively analyzed the correlation between age, disease course, degree of proteinuria, type of immunoglobulin deposits, C3 deposits in glomeruli and renal pathological grade.

**Results:**

(1) 72 HSPN children including 46 boys and 26 girls were included, with a mean age of onset of 9.01 ± 2.65 years old. The majority of these patients (62.5%) had a disease course between 1 week to 1 month. 51 patients presented with mild proteinuria and 21 patients with moderate proteinuria. (2) Renal biopsy results showed that ISKDC Grade IIIa were both predominant in mild proteinuria group (25, 49%) and moderate proteinuria group (11, 52.4%). 32 patients had grade II (44.4%), 2 had grade IIIb (2.8%), 1 had grade IV (1.4%), and 1 had grade VI (1.4%). There was no correlation between age, disease course and renal pathological grade (*p* > 0.05). (3) In patients with mild proteinuria (*n* = 51), 27 (52.9%) HSPN children had a pathological grade ≥ grade III. In patients with moderate proteinuria (*n* = 21), 13 (61.9%) HSPN children had grade ≥ III. There was no significant difference in the proportion of renal pathological grade between the 2 groups (*p *> 0.05). (4) There was no significant correlation between glomerular C3 deposits or immunoglobulin deposit types and renal pathological grade (*p* = 0.776 and *p* = 0.056 respectively).

**Conclusion:**

In HSPN children with mild to moderate proteinuria, longer disease course or heavier urinary protein level is not completely parallel with higher renal pathological grade. ISKDC grade IIIa is the most common pathological grade. Clinicians should pay great attention to the renal injury in patients with mild to moderate proteinuria.

## Introduction

Henoch–Schönlein purpura (HSP), also known as IgA vasculitis since 2012 (1), is characterized with the deposition of IgA-dominant immune complexes in small blood vessels throughout the body affecting mainly skins, joins, gastrointestinal tract and kidney. Henoch–Schönlein purpura nephritis (HSPN) refers to the kidney involvement in HSP and is the most common secondary glomerulonephritis in children. 20–80% of children with HSP occur renal involvement (2), some study even reported an incidence of up to 97% of HSPN within 6 months of the diagnosis of HSP (3). The severity of kidney involvement directly determines the prognosis of the HSP (4–7). 1%–7% of children with HSPN will eventually enter end-stage renal disease (8). Proteinuria is the most common urine abnormality found in HSPN children and has been shown as the primary predictor of long-term kidney survival (9). Renal biopsy with crescentic nephritis (ISKDC grades III–V) have also been reported as predictive of poor prognosis in children with HSPN (10) and is an indication for application of steroids ± immunosuppressant (2). Ideally treatment recommendations for IgA vasculitis should be given based on the severity of urine abnormality, renal function and renal pathology (2, 11). Currently renal biopsy was prevalently recommended in patients presenting with nephrotic proteinuria. But there is much controversy for the renal biopsy indication in HSPN children with non-nephrotic proteinuria. Data on the consistence of urinal protein level and ISKDC grade in HSPN children with mild to moderate proteinuria is limited (12) which results in treatment regimen varied from ACEI or ARB only to steroids ± immunosuppressant among different medical centers, largely relying on the experience of Nephrologists (13). So, we retrospectively analyze the renal pathology in HSPN children with mild to moderate proteinuria and also the association between renal pathologic grade and urinary protein level in this population. Correlation between age of onset, disease course, type of immunoglobulin deposits, presence of C3 deposits in glomeruli and renal pathological grade were also analyzed in this study.

## Patients and research methods

### Patients

HSPN children who were presented with mild to moderate proteinuria and had received renal biopsy without steroid ± immunosuppressant therapy in the Department of Pediatric Nephrology, Second Xiangya Hospital of Central South University between January 2010 and March 2021 were retrospectively included. Informed consent was obtained before renal biopsy from patients' parents. Mild proteinuria is defined by 150 mg < 24 h urinary protein <25 mg/kg and moderate proteinuria is defined by 25 mg/kg ≤ 24 h urinary protein <50 mg/kg (14). The exclusion criteria were as follow: ① Other diseases which needs to be differentiated such as IgA nephropathy, thrombocytopenic purpura, and systemic lupus erythematosus as well as renal damage caused by hepatitis B virus, hepatitis C virus, syphilis, and human immunodeficiency virus (HIV); ② hemorrhagic diseases such as sepsis, diffuse intravascular coagulation, and hemolysis; ③ Receiving drugs that can damage the kidney within 3 months before disease onset. ④ Impaired estimated glomerular filtration rate (eGFR) <90 ml/min/1.73 m^2^. ⑤ Incomplete medical data.

### Research methods

An adequate renal biopsy specimen for light microscopy should contain at least 10 glomeruli. The classification criteria of the International Study of Kidney Diseases in Children (ISKDC) was adopted in grading of renal specimens (15): grade I: minimal glomerular abnormalities; grade II: mesangial proliferation in the absence of crescents; grade III: (a) focal or (b) diffuse mesangial proliferation or sclerosis accompanied by <50% glomerular crescent formation; grade IV: (a) focal or (b) diffuse mesangial proliferation or sclerosis accompanied by 50% to 75% glomerular crescent formation; grade V: mesangial proliferation or sclerosis with crescents in >75% glomeruli; and grade VI: membranoproliferative glomerulonephritis.

Clinical information including age, sex, disease course and 24-hour urinary protein were collected. Disease course is defined as duration from onset of HSP to time of renal biopsy. General characteristics of all patients were described. Associations between age, disease course and renal pathological grades were analyzed. Then, patients were divided into mild and moderate proteinuria group. Renal pathological grades were compared between these two groups. Later, data of renal deposition of IgA, IgG, IgM and C3 were collected. Patients were divided into 4 groups: isolated IgA deposition, IgA + IgG/IgM deposition, IgA + IgG + IgM deposition, and non-IgA/IgG/IgM deposition to explore the association between immunopathologic types and ISKDC grades (16, 17). We also analyzed difference of ISKDC grades between patients with or without renal deposition of C3.

### Statistical analysis

Statistical analysis was performed using the statistical software SPSS 20.0. Count data are expressed as the number of cases and percentage, and measurement data with normal distribution are expressed as the mean ± standard deviation (*x* ± SD). The Pearson *χ*^2^ test was used to compare the differences categorical variables. The Mann–Whitney *U*-test and the Kruskal–Wallis test were used to compare the grade data. A difference of *p* < 0.05 was considered statistically significant.

## Results

### Patient characteristics

A total of 51(70.83%) HSPN children with mild proteinuria and 21(29.17%) with moderate proteinuria were included in our study. There were 46 boys and 26 girls. The mean age was 9.01 ± 2.65 years old. 47(65.3%) cases aged between 5 and 10 years old and 22(30.5%) aged older than 10 years old, only 3(4.2%) cases aged <5 years old. 5 patients (6.9%) had a disease course ≤1 week, 45 patients (62.5%) had a disease course from 1 week to 1 month, 20 patients (27.8%) had a disease course from 1 month to 1 year, and 2 patients (2.8%) had a disease course ≥1 year (as shown in Table 1).

**Table 1:** The general data of patients.

Characteristic	Value
Gender (male/female)	46/26
Age (mean age ± SD, range) (years)	9.01 ± 2.65, 4–15
Age groups	*n* (%)
<5 years old	3 (4.2%)
5-10 years old	47 (65.3%)
>10 years old	22 (30.5%)
Disease course	*n* (%)
≤1 week	5 (6.9%)
1week to 1 month	45 (62.5%)
1 month to 1 year	20 (27.8%)
≥1 year	2 (2.8%)
Proteinuria groups	*n* (%)
Mild proteinuria^a^	51 (70.83%)
Moderate proteinuria^b^	21 (29.17%)
ISKDC^c^ grade	n (%)
II	32 (44.4%)
IIIa	36 (50%)
IIIb	2 (2.8%)
IV	1 (1.4%)
V	0
VI	1 (1.4%)

^a^ Mild proteinuria is defined by 150 mg < 24 h urinary protein <25 mg/kg.

^b^ Moderate proteinuria is defined by 25 mg/kg ≤ 24 h urinary protein <50 mg/kg.

^c^ ISKDC, international study of kidney diseases in children.

### Relationship between age, disease course and renal pathological grade

The most common renal pathological grade for patients with HSPN with mild to moderate proteinuria was grade IIIa (36 patients, 50%), followed by grade II (32 patients, 44.4%), grade IIIb (2 patients, 2.8%), grade IV (1 patient, 1.4%) and grade VI (1 patient, 1.4%) (as shown in Table 1).

To investigate whether renal pathological grade varies among different age and disease course, association between age group, disease course and pathological grades were analyzed. Pathological grades distribution in different age groups and disease courses were shown in Tables 2, 3, respectively. Grade IIIa was predominantly seen in HSPN children aged younger than 5 years old (2, 66.7%) or between 5 and 10 years old (25, 53.2%). Grade II (10, 45.6%) was slightly more common in children older than 10 years old followed by grade IIIa (9, 40.9%). Grade IV and VI were only seen in children older than 10 years old. No grade V was found. Renal pathological grades didn't show significant difference between these three age groups (*p *= 0.879, Table 2).

**Table 2:** Relationship between renal pathological grade and age groups.

Age groups	Renal pathological grade, *n* (%)						Total
	II	IIIa	IIIb	IV	V	VI	
<5 years old	1 (33.3%)	2 (66.7%)	0	0	0	0	3 (4.2%)
5–10 years old	21 (44.7%)	25 (53.2%)	1 (2.1%)	0	0	0	47 (65.3%)
>10 years old	10 (45.6%)	9 (40.9%)	1 (4.5%)	1 (4.5%)	0	1 (4.5%)	22 (30.5%)
Total	32 (44.4%)	36 (50%)	2 (2.8%)	1 (1.4%)	0	1 (1.4%)	72 (100%)

*H* = 0.257, *p* = 0.879.

**Table 3:** Relationship between renal pathological grade and disease course.

Disease course	Renal pathological grade, *n* (%)						Total
	II	IIIa	IIIb	IV	V	VI	
≤1 week	2 (40%)	3 (60%)	0	0	0	0	5 (6.9%)
1-week to 1-month	23 (51.5%)	20 (44.5%)	1 (2.2%)	0	0	1 (2.2%)	45 (62.5%)
1-month to 1-year	7 (35%)	11 (55%)	1 (5%)	1 (5%)	0	0	20 (27.8%)
≥1 year	0	2 (100%)	0	0	0	0	2 (2.8%)
Total	32 (44.4%)	36 (50%)	2 (2.8%)	1 (1.4%)	0	1 (1.4%)	72 (100%)

*H* = 2.903, *p* = 0.407.

As mentioned in patient characteristics, disease course of 1week to 1 month was most commonly seen among patients in our study, consisting of 23 cases of grade II (51.1%), 20 cases of grade IIIa (44.5%), 1 case of (2.2%) grade IIIb and 1case of (2.2%) grade VI. In children with ≤1 week disease course, there were only grade II (2, 40%) and grade IIIa (3, 60%). Among children who had a disease course between 1 month to 1 year, 11 (55%) cases were grade IIIa and 7 (35%) were grade II. 1 (5%) case of grade IIIb and grade IV. Grade IIIa is the exclusive pathological grade seen in children with disease course ≥1 year (2, 66.7%). There was no significant difference of renal pathological grades between HSPN children with different disease courses (*p *= 0.407, Table 3).

### Relationship between severity of urinary protein and renal pathological grade

**Figure 1:**
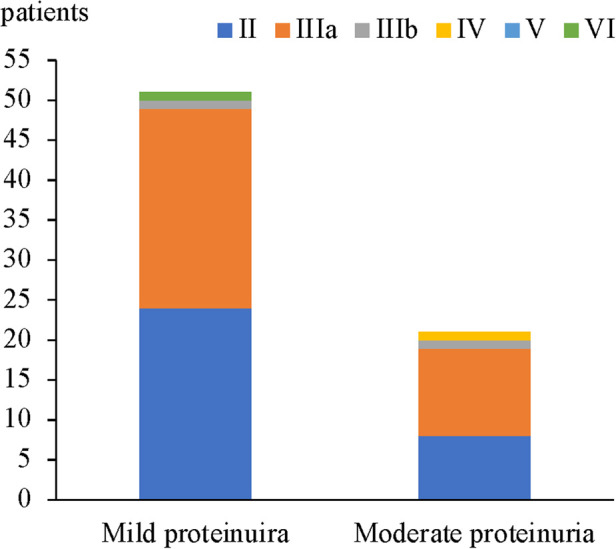
Renal pathological grades in the mild proteinuria and moderate proteinuria groups.

In 51 patients with mild proteinuria, the most common renal pathological grade was grade IIIa (25 patients, 49%), followed by grade II (24 patients, 47%), grade IIIb (1 patient, 2%), and grade VI (1 patient, 2%). In 21 patients with moderate proteinuria, grade IIIa was also the most common (11 patients, 52.4%), followed by grade II (8 patients, 38.1%), grade IIIb (1 patient, 4.8%), and grade IV (1 patient, 4.8%). No significant difference in the proportion of renal pathological grades was found between HSPN children who presented with mild proteinuria and moderate proteinuria (Figure 1 and Table 4).

**Table 4:** Comparison of renal pathological grades between the mild proteinuria and moderate proteinuria groups.

24-h quantitative urinary protein	Renal pathological grade, *n* (%)						Total
	II	IIIa	IIIb	IV	V	VI	
Mild	24 (47%)	25 (49%)	1 (2%)	0	0	1 (2%)	51 (70.8%)
Moderate	8 (38.1%)	11 (52.4%)	1 (4.8%)	1 (4.8%)	0	0	21 (29.2%)
Total	32 (44.4%)	36 (50%)	2 (2.8%)	1 (1.4%)	0	1 (1.4%)	72 (100%)

*U* = 474, *Z* = −0.859, *p* = 0.391.

**Figure 2:**
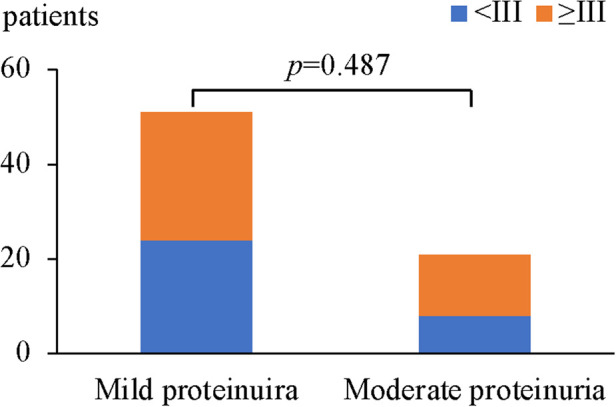
Comparison of renal pathological grades ≥III between the wild proteinuria and moderate proteinuria group.

In HSPN children with mild proteinuria, 24 patients (47.1%) were < grade III, while 27 patients (52.9%) with renal pathological grades of III or higher. Among HSPN children with moderate proteinuria, 8 patients had renal pathological grades < grade III (38.1%), and 13 patients had renal pathological grades ≥ grade III (61.9%) (Figure 2). There was no significant difference in the proportion of patients with renal pathological grade ≥ grade III between the 2 groups (*p *= 0.487, Table 5).

**Table 5:** Comparison of renal pathological grades ≥ grade III between the mild proteinuria and moderate proteinuria groups.

Group	Renal pathological grades, *n* (%)		Total
	<III	≥III	
Mild proteinuria	24 (47.1%)	27 (52.9%)	51 (70.8%)
Moderate proteinuria	8 (38.1%)	13 (61.9%)	21 (29.2%)
Total	32 (44.4)	40 (55.6%)	72 (100%)

*p* = 0.487*, x*^2 ^*= *0.484.

### Relationship between C3 deposition, types of immunoglobulin deposition and renal pathological grade

It has been shown that proportion of grade IIIb to grade VI was relatively higher among patients with IgA + IgG + IgM deposition in glomeruli and IgA + IgM deposition than IgA deposition only (17). To testify whether this is true. We analyzed the difference of renal pathological grades between children with IgA renal deposition only, IgA + IgG/IgM, and IgA + IgG + IgM, and children who were negative for IgA + IgG + IgM deposition.

**Figure 3:**
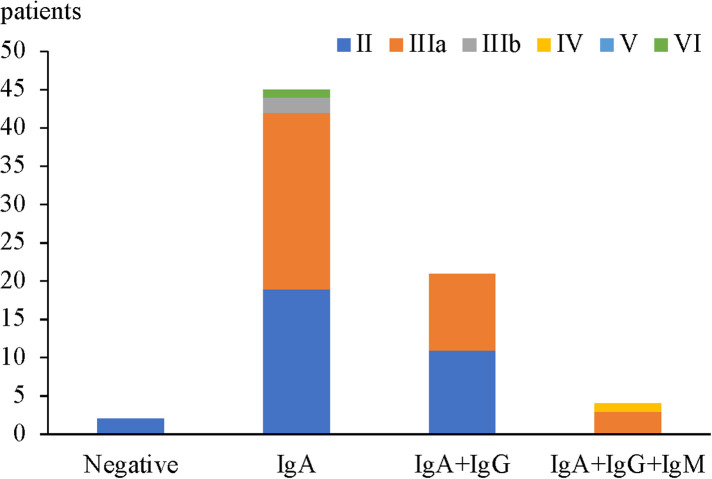
Renal pathological grades for different types of immunoglobulin deposition.

In children with HSPN with mild to moderate proteinuria, only 2 cases were all negative for IgA + IgG + IgM deposition in glomeruli and both were with renal pathological grade II (2 patients, 100%). 45 cases had isolated IgA deposition in glomeruli among which the most common renal pathological grade was grade IIIa (23 patients, 51.2%), followed by grade II (19 patients, 42.2%), grade IIIb (2 patients, 4.4%) and grade VI (1 patient, 2.2%). Among 21 patients with IgA + IgG/IgM deposition, the most common renal pathological grade was grade II (11 patients, 52.4%), followed by grade IIIa (10 patients, 47.6%). Among 4 patients with IgA + IgG + IgM deposition, the most common renal pathological grade was grade IIIa (3 cases, 75%), and the most severe grade was grade IV (1 patient, 25%) (Figure 3). There was no significant correlation between renal pathological grade and type of immunoglobulin deposition (*p* = 0.056, Table 6).

**Table 6:** Relationship between renal pathological grade and different types of immunoglobulin deposition.

Immune complex	Renal pathological grade, *n* (%)						Total
	II	IIIa	IIIb	IV	V	VI	
Negative	2 (100%)	0	0	0	0	0	2 (2.8%)
IgA	19 (42.2%)	23 (51.2%)	2 (4.4%)	0	0	1 (2.2%)	45 (62.5%)
IgA + IgG/IgM	11 (52.4%)	10 (47.6%)	0	0	0	0	21 (29.2%)
IgA + IgG + IgM	0	3 (75%)	0	1 (25%)	0	0	4 (5.5%)
Total	32 (44.4%)	36 (50%)	2 (2.8%)	1 (1.4%)	0	1 (1.4%)	72 (100%)

*H* = 7.568, *p* = 0.056.

**Figure 4:**
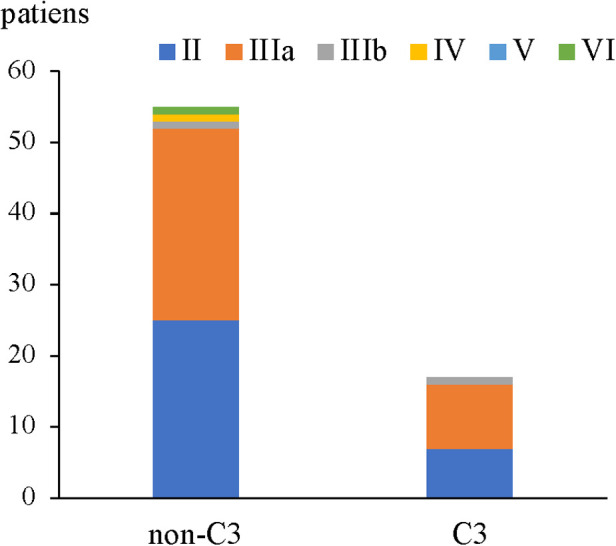
Renal pathological grades for patients with or without glomerular C3 deposition.

Meanwhile, our results showed that 55(76.4%) patients had no glomerular deposition of C3 (Figure 4) in HSPN patients with mild to moderate proteinuria, and no significant difference in term of ISKDC grades was found in patients with or without C3 deposition (*p* = 0.776, Table 7).

**Table 7:** Relationship between renal pathological grade and C3 deposition.

Immune complex	Renal pathological grade, *n* (%)						Total
	II	IIIa	IIIb	IV	V	VI	
Non C3	25 (45.5%)	27 (49.1%)	1 (1.8%)	1 (1.8%)	0	1 (1.8%)	55 (76.4%)
C3	7 (42.2%)	9 (52.9%)	1 (5.9%)	0	0	0	17 (23.6%)
Total	32 (44.4%)	36 (50%)	2 (2.8%)	1 (1.4%)	0	1 (1.4%)	72 (100%)

*Z* = −0.284, *p* = 0.776.

## Discussion

HSPN is the most common secondary glomerulonephritis in children. The overall predominant age of HSPN in children was reported to be 4–9 years old (18, 19). However, data on peak age of non-nephrotic proteinuria in HSPN children is limited and of controversy. Hennies et al. enrolled 202 pediatric HSPN patients from 26 centers in German and found HSPN with a more insidious onset of non-nephrotic proteinuria in children above 10 years old (20). But in Kallash's study from Japan, the peak age of HSPN children with non-nephrotic proteinuria is 7 years old and 9 years old for those with nephrotic syndrome (21). Our study consists with Kallash's study. 69.5% of the study patients aged below 10 years old with a mean age of 9.01 years old. Males were slightly more commonly affected than female with a male to female ratio of 1.77 : 1 which is similar with data reported in the literature (19). It has been reported that the risk of renal involvement in patients increases with age and that the disease is more likely to progress to HSPN in patients over 8 years of age (22–24). In the current study, there were 52 patients with HSPN (72.2%) who were older than 8 years of age. However, we didn't see significantly difference in renal pathological grades among different age groups (<5 years old, 5–10 years old, and >10 years old), suggesting that although the risk of HSPN secondary to HSP increased with age, the severity of renal involvement did not increase accordingly. There are few studies on the correlation between disease course and renal pathological grades. Thus, patients were grouped on the basis of disease course (disease course ≤1 week, 1 week to 1 month, 1 month to 1 year, and ≥1 year). There was no significant difference in renal pathological grades among the groups (*p *> 0.05), suggesting that a longer disease course does not indicate more severe renal pathological changes.

Grade III was the most common ISKDC grade in non-nephrotic proteinuria with or without hematuria in Avramescu et al.'s study. Similarly in our 21 patients with moderate proteinuria, grade IIIa (11, 52.4%) is the most predominant grade. In the 51 children with mild proteinuria, number of grade IIIa (25, 49%) and II (24, 47%) was more close. Together, our results showed that grade IIIa was the most prevalent grade in HSPN children with non-nephrotic proteinuria (36, 50%). followed by grade II (32 patients, 44.4%), grade IIIb (2 patients, 2.8%), grade IV (1 patient, 1.4%) and grade VI (1 patient, 1.4%). There was no patient with renal pathological grade of grade V. However, Luo et al. (16) retrospectively analyzed the clinical an d pathological data of 180 children with HSPN with proteinuria. The results indicated that moderate proteinuria was more predominant (57 patients, 31.7%), followed by massive proteinuria (51 patients, 28.3%), and that the most common renal pathological grade for moderate proteinuria was grade II (31 patients, 54.4%). These differences may be related to different sample size, source of the patients, and timing of renal biopsy in each study. Further comparison of renal pathological grades between HSPN children with mild proteinuria and those with moderate proteinuria was done and there was no significant difference (*p* > 0.05).

Renal pathological grade (ISKDC grade III–VI) is one of the most important factors with regard to a poor prognosis (10). When the crescent is greater than 50%, the incidence of chronic kidney disease progression is 5–20% (25). Also, the renal pathological grades ≥ IIIb in our study patients was quite few. In order to improve the statistical reliability, we considered HSPN children with a renal pathological grade ≥ grade III as an ensemble and made further analysis. There were 27 patients (52.9%) with a renal pathological grade ≥ grade III in the mild proteinuria group and 13 patients (61.9%) with renal pathological grade ≥ grade III in the moderate proteinuria group with no significant difference between groups (*p *= 0.487), suggesting that the urinary protein level was not completely parallel to the renal pathological grade in HSPN children with non-nephrotic proteinuria. Mild proteinuria is not necessarily associated with mild renal pathology. Ye et al. (4) conducted a retrospective study including 694 children with HSPN, the results indicated that there was no significant difference in 24-h quantitative urinary protein between patients with HSPN type I and type IIa (*p* = 0.873), and no significant difference in 24-h quantitative urinary protein (*p* = 0.190) among patients with HSPN IIb, IIIa and IIIb. However, the 24-h quantitative urinary protein for patients in the HSPN I and IIa groups was significantly lower than that for patients in the HSPN IIb, IIIa, and IIIb groups (*p* < 0.01). In another study including 180 pediatric HSPN (16), the differences in pathological grades among the 4 clinical types of proteinuria (micro, mild, moderate and massive) were statistically significant (*p* = 0.002). With the increase in proteinuria severity, the pathological grade showed an increasing trend. The results of the above studies are different from the results of this study, potentially because the data used in this study did not include data for patients with HSPN with severe proteinuria.

Immunoglobulin deposition can stimulate the activation of mesangial cells to produce a large number of cytokines and promote cell proliferation, ultimately causing kidney damage (26). Theoretically, the more types of immunoglobulin deposition, the stronger is the renal inflammatory response, and the more severe is the kidney pathological damage. However, so far, the results of many studies on this aspect are quite different. Previous study conducted by Cheng (17) analyzed the correlation between the immunopathological type and clinical pathology of 62 children with HSPN and found that compared with that in patients with isolated IgA deposition, the proportion of grade IIIb–VI disease among patients with IgA + IgG + IgM deposition and IgA + IgM deposition was relatively higher (*p* < 0.05). But Luo et al. (16) and Qin et al. (27) showed that there was no significant correlation between renal pathological grade and type of immunoglobulin deposition (*p* = 0.056). our results showed similar findings with Luo et al. (16) and Qin et al. (27). 45 cases had isolated IgA deposition in glomeruli among which the most common renal pathological grade was grade IIIa (23 patients, 51.2%), followed by grade II (19 patients, 42.2%). Among 21 patients with IgA + IgG/IgM deposition, the most common renal pathological grade was grade II (11 patients, 52.4%), followed by grade IIIa (10 patients, 47.6%). There was no significant correlation between renal pathological grade and type of immunoglobulin deposition (*p* = 0.056, Table 6) suggesting that the additional deposition of IgM and/or IgG may not contribute to a more severe renal damage.

Activation of complement system has been suggested to be involved in the pathogenesis of HSPN (28, 29). However, serum level of C3, glomerular C3 deposition or glomerular C4d deposition were not shown to be correlated with the clinical or pathological severity of HSPN (16, 30, 31). Our result showed most of HSPN children (76.4%) with mild to moderate proteinuria had no glomerular C3 deposition while Luo et al.'s study showed only 26.1% of 180 HSPN children were absent of glomerular C3 deposition (16). Notably, Luo et al.'s study included 51 patients with nephrotic range proteinuria which could result in the difference of proportion of C3 deposition. Despite all this, both studies didn't find correlation of glomerular C3 deposition with ISKDC grades.

There are some limitations of the current study. First of all, ISKDC is currently the most widely used classification for HSPN and mainly focus on active inflammation which is reflected by crescents formation. Increasing studies argue that the 2016 revised Oxford classification (MEST-C scores) should be recommended for children and adult patients with HSPN (18, 32, 33). Segmental glomerulosclerosis/adhesion (S) and tubular atrophy/interstitial fibrosis (T), which are not emphasized or included in ISKDC classification, are shown to be the prognostic factor for renal outcomes in those studies. We didn't find association of age, disease course, proteinuria level and type of immunoglobulin with ISKDC classification. Association of these factors with T and S lesions is of great value to be further investigated in future study. The second limitation is that ideally, the association between duration of renal involvement and renal pathological changes should be evaluated. However, unlike nephrotic proteinuria, HSPN children with mild to moderate proteinuria are more likely to be lack of clinical manifestation like edema, obvious foamy urine or decreased urine output, resulting in a delayed discovery of urinary protein. First visit of most patients were not in our hospital. Irregular follow-up in partial patients made it harder to acquire exact duration of renal involvement. Thus, association between duration of disease course and ISKDC grades was analyzed instead. Another limitation of this study is the relatively small sample size and also, we did not have the long-term follow-up data on those with renal pathological grades ≥grade III. Therefore, future study will be needed to discuss the optimized time for biopsy and medicine intervention for HSPN children with non-nephrotic proteinuria.

In conclusion, our study showed a longer disease course does not necessarily indicate more severe renal pathological changes and urine protein levels did not parallel with the severity of renal pathological changes in HSPN children with non-nephrotic proteinuria. but the proportion of renal pathological grades ≥grade III can't be neglected. More attention should be paid to the close follow-up of HSPN children with mild to moderate proteinuria. Additionally, in children with HSPN with mild to moderate proteinuria, there was no significant correlation between glomerular C3 deposition or type of immunoglobulin deposition and renal pathological grade.

## Data Availability

The original contributions presented in the study are included in the article/Supplementary Material, further inquiries can be directed to the corresponding author/s.
